# MultiAgency, prospective, exploratory, non-intervention, cohort Study on Human Impact Exposure oNboard high-speed boats (MASHIEN): protocol

**DOI:** 10.1136/bmjopen-2024-090993

**Published:** 2025-05-02

**Authors:** Johan Ullman, Stephen D Myers, Kai-Thorsten Bretschneider, Karen R Kelly, Yann Daniel, Victor Hurpin, Justus Kaehler, Jan Ivar Kåsin, Knut Hveding, Neil Mansfield, Spyros D Masouros, Daniel Perl, Nicole Wijnands, Isabelle Vallee, Veerle Stevens, John J Fraser, Ola Rolfson, Yohan Robinson

**Affiliations:** 1, Swedish Armed Forces Centre for Defence Medicine, Gothenburg, Sweden; 2 Department of Orthopaedics, Sahlgrenska Academy, University of Gothenburg, Goteborg, Sweden; 3 Occupational Performance Research Group, University of Chichester, Chichester, UK; 4 Schifffahrt- und Arbeitsmedizin, Schifffahrtmedizinisches Institut der Marine, Kronshagen, Germany; 5 Warfighter Performance, Naval Health Research Center, San Diego, California, USA; 6 Paris Fire Brigade, Paris, France; 7 Military Training Hospital Clermont-Tonnerre, Brest, France; 8 Flymedisinsk Institutt, Oslo, Norway; 9 The Norwegian Sea Rescue Society, Oslo, Norway; 10 Nottingham Trent University, Nottingham, UK; 11 Department of Bioengineering, Imperial College London, London, UK; 12 Uniformed Services University of the Health Sciences, Bethesda, Maryland, USA; 13 Hoofd Sociaal Medische Dienst, Den Helder, The Netherlands; 14 Canadian Armed Forces Health Support and Casualty Services, Ottawa, Ontario, Canada; 15 Queen Astrid Military Hospital, Brussels, Belgium; 16 Department of Physical Medicine and Rehabilitation, Uniformed Service University for Health Sciences School of Medicine, Bethesda, Maryland, USA; 17 Sports Medicine Research Institute, University of Kentucky, Lexington, Kentucky, USA; 18 Capio Spine Center Gothenburg, Gothenburg, Sweden; 19 Centre for Disaster Medicine, University of Gothenburg, Gothenburg, Sweden

**Keywords:** Back pain, OCCUPATIONAL & INDUSTRIAL MEDICINE, Risk management, Adult orthopaedics, Orthopaedic sports trauma

## Abstract

**Introduction:**

High-speed boat operations expose personnel to slamming-induced impacts, which can lead to musculoskeletal injuries and cognitive impairments. Despite existing safety measures, regulations and protocols, the risk of injuries remains significant. The MultiAgency, prospective, exploratory, non-intervention, cohort Study on Human Impact Exposure oNboard high-speed boats study aims to investigate the nature and magnitude of these impacts, their acute and long-term health effects, and potential injury prevention strategies to improve operational safety and performance.

**Methods and analysis:**

This is an ongoing multicentre, prospective, non-intervention, observational cohort study. The first participant was enrolled on 23 August 2024. High-speed boat operators log self-reported pain data via a smartphone app, using a Visual Analogue Scale and pain drawings. Triaxial accelerometers are installed on boat hulls and worn by participants to measure impact exposure. Data analysis assesses correlations between exposure and reported pain, enabling the identification of risk factors and the development of safety guidelines for high-speed boat operations.

**Ethics and dissemination:**

The study has received ethical approval from the relevant ethics committees, including the Swedish Ethics Review Authority (no. 2022-04931-01). All participants will provide informed consent before enrolment. The findings will be disseminated through technical reports, articles in peer-reviewed journals, conference presentations and direct engagement with military and maritime stakeholders to enhance training protocols and safety measures.

**Trial registration number:**

NCT05299736.

**STRENGTHS AND LIMITATIONS OF THIS STUDY**

- This study is a multicentre, prospective cohort design, allowing for a comprehensive evaluation of human impact exposure across diverse operational environments.
- The use of objective impact measurement through triaxial accelerometers enhances the accuracy and reliability of exposure data.
- The inclusion of self-reported pain assessments provides valuable subjective data on the effects of impact exposure, although it may be susceptible to recall bias.
- The study population is limited to professional high-speed boat operators, which may restrict the generalisability of findings to recreational users or other occupational groups.
- The observational nature of the study means that causality cannot be definitively established between exposure and health outcomes, necessitating further interventional research for validation.

## Introduction

 High-speed boat operations expose humans to slamming-induced impacts, where the boat hull hits the waves, transmitting significant impact forces through the boat to its occupants. The intensity and frequency of slamming impacts depend on a variety of factors, including the speed of the boat, the sea state and the design of the boat.^1^ Musculoskeletal injuries sustained during high-speed boat operations are typically caused by repeated exposure to whole-body impacts.^1 2^ The induced forces can bend, twist and compress the spine, causing vertebral fractures, disc herniations and distortion injuries. Slamming can also cause significant impact forces transmitted through the upper extremities, leading to injuries such as rotator cuff tears and wrist fractures.^3^ Moreover, these forces may affect the brain, leading to neurological symptoms and injuries, including cognitive effects and mild traumatic brain injury.^4^

Several studies have evaluated the effects of impact exposure on operator performance during high-speed boat transits.^5^ Post-transit, operators were found to have decreased running performance,^6^ increased inflammation as reflected by salivary cortisol^7^ and increased muscle damage as reflected by elevated levels of creatine kinase.^8^ Impaired cognitive functionality affecting operational readiness, even unconsciousness, has been reported.^1^ A recently published systematic review identified five scientific reports describing 3312 injuries sustained during 3467 person-years onboard high-speed boats.

Repetitive exposure to slamming may lead to chronic musculoskeletal disorders, pain conditions and disabilities. Long-term exposure to whole-body vibration is known to have detrimental cumulative effects on the intervertebral discs and increases the incidence of low-back pain.^9 10^ Additionally, slamming impacts can cause musculoskeletal injuries, particularly to the spine and lower extremities.^13 5 11^,^14^ To address the occurrence of these injuries, legal exposure regulations are in place, and operational restrictions have been implemented.^15^ However, littoral and amphibious operations often require rapid transits to transport operators, irrespective of weather conditions and sea states.

There are several reasons why human impact exposure onboard high-speed boats needs to be studied.

- *Injury prevention*: researchers can identify effective strategies to prevent or mitigate injuries by examining the nature and severity of slamming impacts and their correlation to reported pain and risk of musculoskeletal injuries due to repeated exposure.^116^,^18^
- *Health and safety regulations*: understanding the nature and magnitude of impact exposure that may cause injuries can guide the development of relevant health and safety regulations for high-speed boat operations and help avoid unwarranted operational restrictions.^19^
- *Boat design and technology*: by studying impact exposure, researchers can identify areas for improved boat design and technology.^20^ For example, by developing better shock-absorbing materials or hull shapes, naval architects can help reduce the exposure to impact on the human body.
- *Training*: exposure to high, potentially harmful magnitudes of whole-body impacts has not been shown to have positive training effects. Knowing what magnitudes of exposure are sustainable can improve training routines and objectives.
- *Operational optimisation*: the slamming-induced impacts can also affect the performance of high-speed boats.^21^ By studying the nature and severity of slamming impacts, researchers can identify ways to optimise boat performance, such as adjusting speed or altering course in response to changing sea conditions. With a better understanding, operators can make better decisions, improving operational efficiency.

This prospective, multicentre, observational, cohort study on human impact exposure onboard high-speed boats aims to determine (1) the nature and the magnitudes of slamming-induced impacts on humans onboard and (2) the types and magnitudes of impacts that are potentially harmful.

## Methods and analysis

### Study design, setting and participants

This prospective, self-controlled, non-intervention, multicentre cohort study investigates the acute effects of slamming-induced impact exposure on high-speed boat operators’ musculoskeletal systems and cognitive functions. The study protocol is reported according to Strengthening the Reporting of Observational Studies in Epidemiology guidelines and was registered prospectively on ClinicalTrials.gov with the identifier NCT05299736.^22^ This study has been designed by the North Atlantic Treaty Organization (NATO) Science & Technology Organization, Research Task Group HFM-344. The recruitment of academic centres started in 2022 and the first participant was enrolled on 23 August 2024. We expect to close the inclusion of study participants before 30 June 2027. The schedule of participant enrolment, exposures and assessments is presented in table 1.

**Table 1:** Schedule of enrolment, exposures and assessments

Timepoint	Study period							
	Enrolment	Allocation	Post allocation					Close-out
	*−t_1_*	0	*t_1_*	*t_2_*	*t_3_*	*t_4_*	*etc*	*t_90_*
Enrolment:								
Eligibility screen	X							
Informed consent	X							
Allocation		X						
Exposure:								
Slamming forces			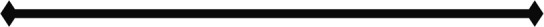					
Whole body impacts			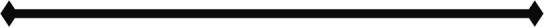					
Assessments:								
Questionnaire		X						X
Level of pain (VAS)		X	X	X	X	X	X	X
Pain character (pain drawing)		X	X	X	X	X	X	X

Timepoints *t* define days 1–90.

VAS, Visual Analogue Scale.

Eligible participants are military and civilian agencies, organisations and institutions that operate boats capable of hydroplaning speeds, have professional high-speed boat operators and allow the installation of accelerometer devices and data recording. The study will include operators in each centre between age 18 and 65 years who either operate or are being regularly transported onboard high-speed boats and have signed the informed consent form.

### Outcomes, data sources and measurements

#### Pain

Pain is a common symptom experienced by high-speed boat operators exposed to repeated slamming impacts, and it is a common reason that operators seek medical attention or modify their work activities.^1^ By studying the occurrence and duration of pain and its correlation in time after slamming-induced impacts, the study aims to assess the risks of injurious exposure. Pain can be monitored non-invasively using validated methods, such as the Visual Analogue Scale (VAS) or Numeric Rating Scale, which rate pain intensity, and pain drawings to assess pain location, duration and interference with activities.

**Figure 1:**
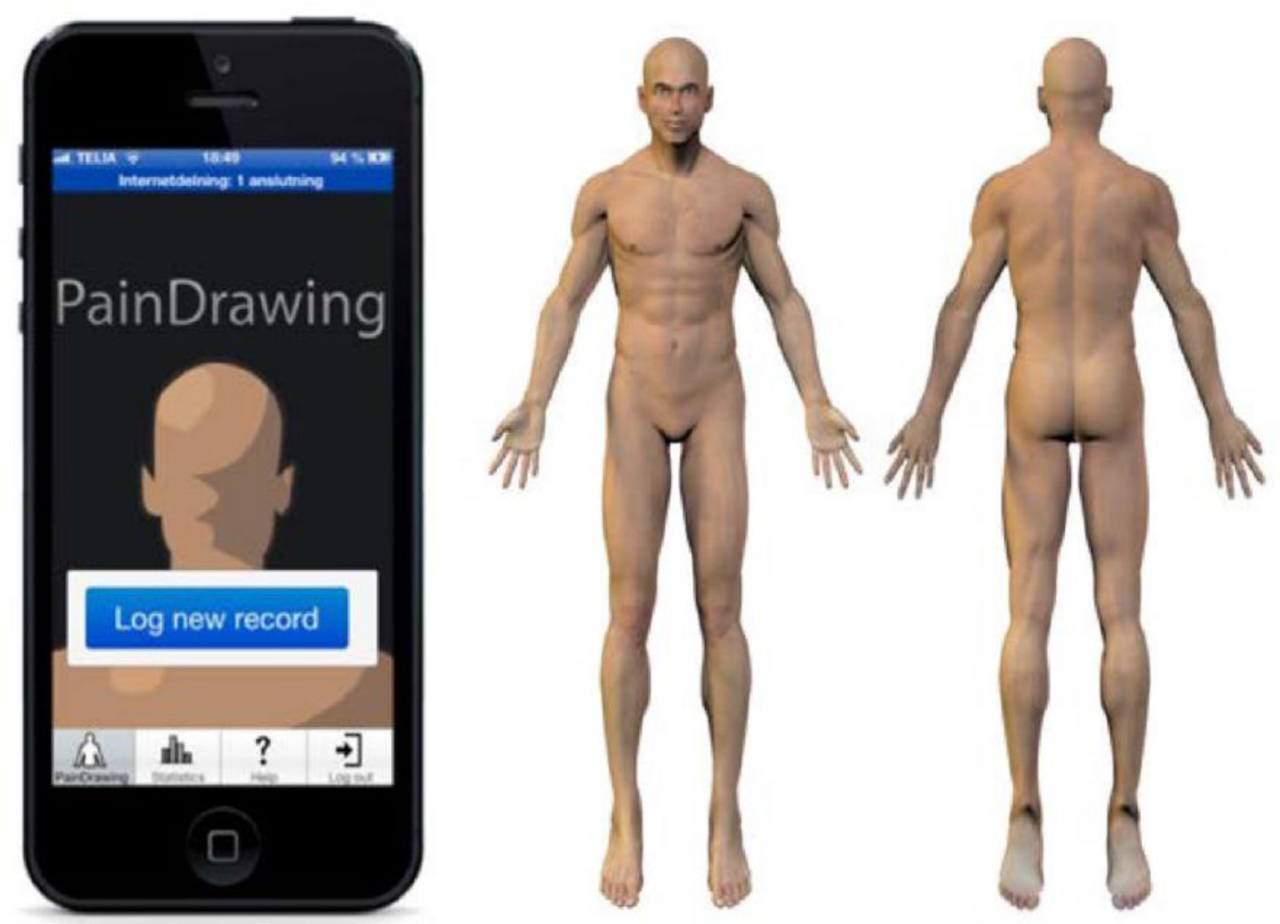
The PainDrawing application allows registering individual pain levels using the Visual Analogue Scale and a standardised pain drawing for pain location and characteristics.

In this study, self-reported pain will be logged daily through a smartphone application, PainDrawing (figure 1),^23^ adapted specifically for this project.^24^ The application is based on the Nordic Minister Council’s pain drawing form and a VAS. PainDrawing can be downloaded freely from Google Play and Apple AppStore and works offline and in flight mode. Reported pain data will comprise the following characteristics: location, pain modality, intensity and duration. Participants will be urged to log pain daily for the whole test period, regardless of whether they have been on board a boat or not. Participants will serve as their own controls by comparing periods of exposure onboard with periods of not working onboard.

#### Exposure to impacts

**Figure 2:**
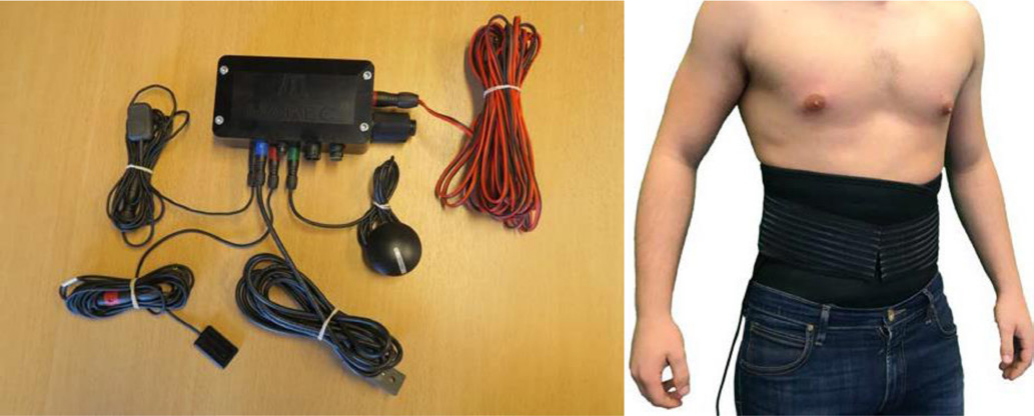
On the left is a photograph of the MAREC device and related connections and on the right the kidney belt containing a three-axis accelerometer worn by the participating operators.

A data logger system (MAREC, Research Electronics AB, Siljansnäs, Sweden) was designed specifically for this study. The device comprises three triaxial micro-electromechanical system (MEMS) accelerometers with a range of ±25*g*, a Global Positioning System antenna for tracking speed, and a data logger unit with 10 digital channels (figure 2). The acceleration data are recorded at a sampling rate of 600 Hz and stored on an internal removable 16 GB USB memory stick. One accelerometer fitted to the hull will record hull accelerations. Two accelerometers fitted in kidney belts, as depicted in figure 2, will be worn by two operators, normally the coxswain and the navigator.^11^

Accelerations will be analysed on all nine channels for the following characteristics:

- *Number of impacts*: slamming impacts will be defined by the amplitude of the vertical accelerations measured on the boat hulls, where this peak acceleration exceeds 20 m/s^2^ or 2*g*. Peak accelerations below this level will not qualify as impacts relevant for analysis in this study.
- *Peak acceleration levels*: the peak acceleration value will be defined as the highest registered acceleration data point in each impact.
- *Range of rise times and correlation to peak acceleration levels*: rise time is defined as the time from nominal 0*g* to peak acceleration value.
- *Range of vector sum*: the force vectors will be the calculated vector sum resulting from the vertical force vector Z and the two horizontal force vectors X and Y. This value will be higher than any of the included three single-axis values and will show the magnitude of the total impact.
- *Three-dimensional impact measurement*: the direction and the acceleration of each impact will be recorded on the hull and on two boat operators per boat, using three-axis accelerometers.
- *Range of impact durations*: impact durations will be calculated as the time from the datapoint where the vertical acceleration exceeds 0*g* in each hull impact, exceeding 2*g* vertically until the last datapoint falls under 1.0*g*.
- *Range of jerk (speed of onset of impacts)*: the range of jerk, defined as the rate of the initial change of acceleration, will also be analysed. It is the derivative of the peak acceleration value (m/s^2^) divided by the time it took to reach it.^25^

**Figure 3:**
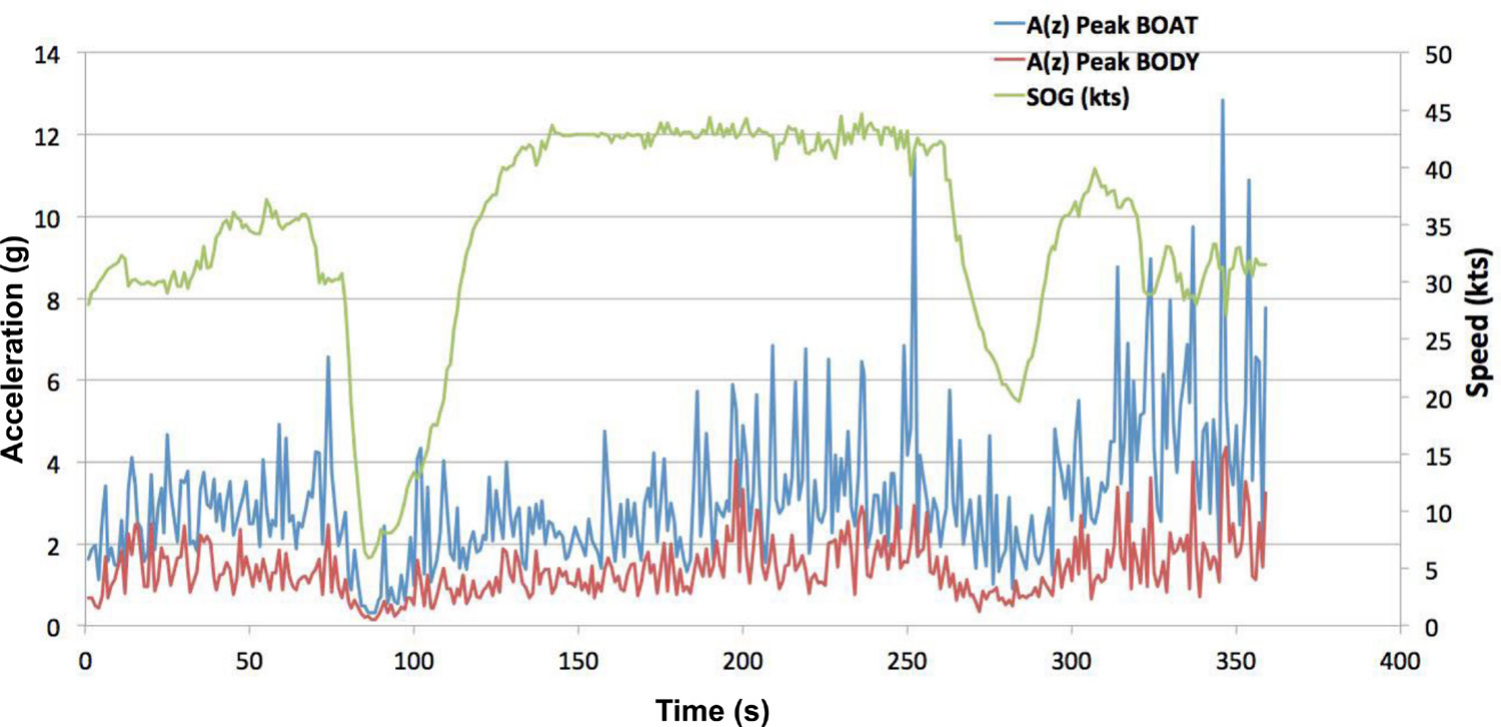
Example of the impact exposure data generated by the MAREC. The above example shows the time (400 s) on the x-axis. On the left y-axis is acceleration measured in *g*=9.81 m/s^2^, with red for the operator and blue for the boat hull, and on the right y-axis is the boat speed measured in knots shown by the green line. SOG, speed over ground.

**Figure 4:**
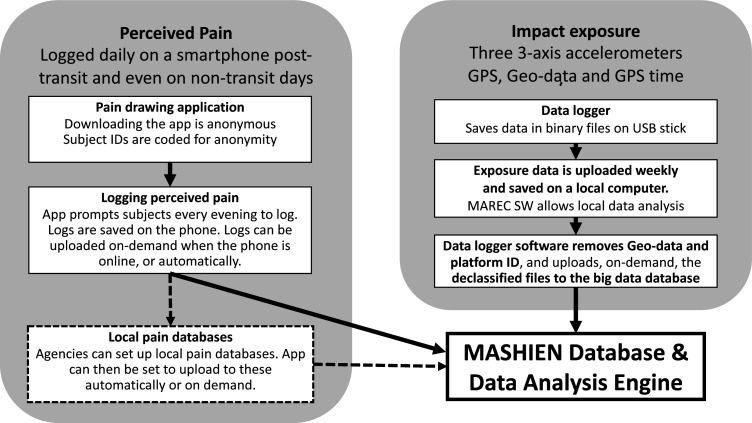
Flow of exposure data and pain data. All agencies will have access to the exposure data they have submitted to the MultiAgency, prospective, exploratory, non-intervention, cohort Study on Human Impact Exposure oNboard high-speed boats (MASHIEN) Database and to the anonymised pain data from their units. Agencies wishing to also save anonymised pain data locally may set up local databases for this purpose. GPS, Global Positioning System. MAREC SW, MAREC accelerometer recorder software.

Data output from the device will be presented in various graphic formats, for example, line graphs, allowing zooming from one full day of exposure down to showing the shape of a single impact regarding all characteristics of the impact (figure 3), or as histograms showing the numbers of impacts reaching different peak levels.^26^ To allow for a wide variety of analyses, all acceleration data will be stored in binary files as unfiltered raw data (figure 4). If a participating centre cannot use the MAREC data logger, another device with identical performance, sensors, software and output file format may be used.

#### Sea state and meteorological data

Data will be collected on weather and sea state from meteorological sources as these factors affect the amount of slamming. Oceanographical data (sea state) and meteorological data (wind, temperature, rain) will be extracted from global meteorological databases. Even the characteristics of the boat type used will be registered while mounting the device onboard.^11^

#### Health and working conditions survey

A validated web-based questionnaire for surveying the health and working conditions of high-performance marine craft populations will be designed to assess a range of factors, including musculoskeletal injuries, noise exposure, psychological stress and fatigue.^12^

### Target sample size

A recent systematic review of five studies reported 1.1 injuries per person per year serving as high-speed boat operators.^5^ Our study follows operators for at least 2 months, estimating that one of six operators may experience one acute episode with musculoskeletal injury. The Minimally Clinically Important Difference for back pain VAS is 14 mm, and the SD is 20 mm.^27^ Consequently, a minimum number of 192 participants would be necessary to reach 80% power (1 minus type II error probability) at a 0.05 significance level (type I error probability) if followed for 2 months.

### Statistical methods

We will report descriptive statistics and calculate the mean, SD and range for human impact exposure and pain scores. Peak acceleration values, m/s^2^, will be registered in all three axes, x, y and z. A data analysis engine will categorise impacts with various combinations of the impact characteristics and allow for calculating correlations to registered pain experiences and various variables of the reported pain. We will use Pearson correlation coefficients to examine the association between human impact exposure and pain scores. We will also use linear regression to examine these associations while controlling for potential confounding variables (eg, age, sex, body mass index). Multivariate analysis based on analysis of variance (ANOVA) and logit models will be used to identify which characteristics of the impacts correlate to reported pain of various intensities, locations and durations after exposure. The results of the statistical analysis are expected to indicate which impact characteristics show a correlation to reported experiences of pain. Based on these findings, we will establish which impact exposure levels and characteristics are safe regarding physical and cognitive capabilities.

This analysis can also validate alternative ways of quantifying human exposure to discrete whole-body impacts and their potential relevance for predicting the risk of injury. Candidates for alternative units include calculated kinetic energy transferred or absorbed, J/kg, and the speed of this transfer, mechanical power, J/(kg·s). The analysis may need to include the calculated total change of speed and the distance during which the retardation of the boats and the human bodies occurs in each impact.

#### Subgroup analysis

We will conduct subgroup analyses to explore differences in the association between human impact exposure and pain scores among different populations (eg, operators vs passengers), boat hull types and participant subgroups. As the data remain with the participating centres, exposure may be further compared between subgroups such as boat types, units, locations, missions and operators.

#### Risk of bias, confounding factors and compliance

Cohort studies have several sources of bias, including selection bias, attrition bias and measurement bias. This study aims to apply the available measures to minimise bias, such as ensuring representative sampling, using objective measures of exposure and outcome, conducting follow-up assessments in a standardised manner and adjusting for potential confounders in the analysis.

The use of self-reported measures, such as questionnaires or surveys, to assess health status among high-speed boat operators is subject to bias and limitations, including the potential for dissimulation or inaccurate reporting. Therefore, it is essential to use multiple methods and sources of information to accurately assess the health status and occupational hazards faced by high-speed boat operators. Regarding pain as a measure of outcome, some studies identified dissimulation as a cause of measurement bias.^13^ Dissimulation may be more common among military populations, including military high-speed boat operators, due to concerns about the impact of reported health issues on job duties and career advancement.^1 13^ However, other studies have found that dissimulation rates among military personnel are generally low.^28^

Possible confounders of recorded pain are injury events unrelated to impact onboard high-speed boats. These can be related to other occupational activities (ie, parachuting) or leisure activities (ie, exercise and sports).^13^ Therefore, the pain log application will ask for information in free text about relevant onboard events and outside work activities. Pre-existing pathologies could also confound the results (ie, history of lumbar pain or radiculopathy), even though they may be related to injuries onboard high-speed boats before inclusion. Therefore, the baseline health status will be determined using the questionnaire developed by de Alwis *et al*^12^

There is also a risk of reduced compliance in logging pain from those not experiencing any pain. To boost compliance, participants can be told that a response rate of at least 24 days per month is required to be considered having complied. As the participants are generally interested in the occupational health hazards being investigated, a fair compliance rate can be expected.

### Patient and public involvement

This study was designed with the involvement of retired high-speed boat operators and the involvement of the multinational naval operator community.

## Ethics and dissemination

This study has received ethical approval from the relevant institutional review boards and ethics committees, including the Swedish Ethics Review Authority (no. 2022-04931-01). All participants provide informed consent before enrolment, ensuring they understand the study’s objectives, procedures, potential risks and benefits. Confidentiality and data protection will be maintained following the General Data Protection Regulation and applicable national laws. Personal identifiers will be removed from the dataset before analysis to ensure participant anonymity.

The results of this study will be published as a NATO Science & Technology Organization technical report, and disseminated through multiple channels, including peer-reviewed scientific journals, international conferences and direct presentations to military and maritime stakeholders. Findings will also be shared with relevant governmental and non-governmental organisations to influence policy and operational guidelines. Open-access data summaries will be made available where possible to promote transparency and further research in occupational health and safety for high-speed boat operators. Additionally, participating organisations will receive tailored reports to support evidence-based improvements in training, equipment and operational procedures.

## Discussion

This study provides a comprehensive approach to evaluating human impact exposure onboard high-speed boats. By incorporating objective measurement tools such as triaxial accelerometers and self-reported pain assessments, it aims to identify risk factors and inform future safety measures. However, several limitations must be considered when interpreting the findings.

One key limitation is the observational nature of the study, which prevents definitive conclusions about causality between impact exposure and musculoskeletal injuries. While correlation analyses will be conducted, future interventional studies are necessary to confirm causal relationships. Additionally, the study relies on self-reported pain assessments, which are subject to recall bias and individual pain perception variations, potentially leading to inconsistencies in data reporting.^29^

The study population is restricted to professional high-speed boat operators, limiting the generalisability of findings to other groups such as recreational users or operators in different occupational settings. Furthermore, differences in operational environments, including boat types, sea conditions and mission profiles, may introduce variability in exposure levels, making it challenging to standardise impact measurement across all study sites.

Confounding variables such as pre-existing musculoskeletal conditions, overall physical fitness and variations in operator experience could influence the outcomes despite efforts to control for these factors.^30^ Additionally, compliance with daily pain logging through the smartphone application may fluctuate, leading to potential gaps in data collection that could affect the robustness of findings.

Another limitation is the potential impact of national and organisational differences in health and safety regulations. Since multiple countries and agencies are involved, variations in operational protocols and exposure limits may affect how findings are interpreted and applied to different regulatory frameworks.

At last, while the use of accelerometers provides precise impact exposure data, the interpretation of biomechanical forces and their effects on human physiology remains complex. Future research should aim to refine exposure thresholds and explore biomechanical modelling to better predict injury risks.

Despite these limitations, this study represents a significant step forward in understanding the occupational hazards associated with high-speed boat operations. Its findings can inform evidence-based guidelines for injury prevention, contribute to safer operational practices and support improvements in boat design and suspension technology. Data on what magnitudes and characteristics of impacts correlate to reported pain can indicate what levels and types of exposure are potentially harmful. This knowledge can be used to suggest limits for impact exposure during training.

The exposure results can help validate the scope and the severity of this problem and assess if preventive measures are needed or justified. The availability of these data can help establish the clinical and economic burden of these injuries and inform decision-makers regarding resource allocation, compensation and other relevant issues. Finally, the results can guide the development of evidence-based guidelines and standards for the safe operation of high-speed boats. This can reduce the risk of injuries and promote the health and well-being of high-speed boat operators.

Based on the data generated by this study, dashboard instruments can be calibrated for each type of boat to give boat operators continuous real-time information about when hull impacts rise to dangerous levels. Such devices need only a single three-axis hull accelerometer and a visual display.^31^ Additional research can be aimed at demonstrating the effectiveness of this type of device in real-world use and operations. Further research will investigate whether the results of this study are transferable to human impact exposure in all-terrain vehicles^32 33^ and could identify occupationally relevant exposure limits for off-road and all-terrain vehicles.
