# Supplementary material for: MultiAgency, prospective, exploratory, non-intervention, cohort Study on Human Impact Exposure oNboard high-speed boats (MASHIEN): protocol
Source: BMJ Open. 2025 May 2;15(5):e090993. doi: 10.1136/bmjopen-2024-090993 (PMC12049913; doi:10.1136/bmjopen-2024-090993)
Supplement: Supplementary file 1 [file bmjopen-2024-090993.reviewer_comments.pdf]

PEER REVIEW HISTORY

BMJ Open publishes all reviews undertaken for accepted manuscripts. Reviewers are asked to complete a checklist review form and are provided with free text boxes to elaborate on their assessment. These free text comments are reproduced below.

ARTICLE DETAILS

Title (Provisional)

Protocol for a Multi-Agency, prospective, exploratory, non-intervention, cohort Study on Human Impact Exposure oNboard high-speed boats (MASHIEN)

Authors

Ullman, Johan; Myers, Stephen D.; Bretschneider, Kai-Thorsten; Kelly, Karen R.; Daniel, Yann; Hurpin, Victor; Kaehler, Justus; Kåsin, Jan Ivar; Hveding, Knut; Mansfield, Neil; Masouros, Spyros D.; Perl, Daniel; Wijnands, Nicole; Vallee, Isabelle; Stevens, Veerle; Fraser, John J.; Rolfson, Ola; Robinson, Yohan

---

VERSION 1 - REVIEW

**Reviewer**	**1**
**Name**	**Catapano, Michael**
**Affiliation**	**Harvard University**
**Date**	**09-Jan-2025**
**COI**	**None**

This a very well thought out and written protocol.

No Concerns.

**Reviewer**	**2**
**Name**	**Tiwald, Gerhard**
**Affiliation**	**Zealand University Hospital Koge, Emegency Department**
**Date**	**04-Feb-2025**
**COI**	**None**

Interresting topic and well designed protocol. Looking forward to the results.

VERSION 1 - AUTHOR RESPONSE

Reviewer 1: Dr. Michael Catapano, Harvard University

Comment:
"This is a very well thought out and written protocol. No concerns."

Response:
Thank you for your positive feedback. No changes were required based on your comments.

Reviewer 2: Dr. Gerhard Tiwald, Zealand University Hospital Koge

Comment:
"Interesting topic and well-designed protocol. Looking forward to the results."

Response:
We appreciate your positive evaluation and enthusiasm for our study. No changes were requested, but we have strengthened certain aspects based on the editorial team’s comments.
